# Exploring the Applicability of Using Natural Language Processing to Support Nationwide Venous Thromboembolism Surveillance: Model Evaluation Study

**DOI:** 10.2196/36877

**Published:** 2022-08-05

**Authors:** Aaron Wendelboe, Ibrahim Saber, Justin Dvorak, Alys Adamski, Natalie Feland, Nimia Reyes, Karon Abe, Thomas Ortel, Gary Raskob

**Affiliations:** 1 Department of Biostatistics and Epidemiology Hudson College of Public Health University of Oklahoma Health Sciences Center Oklahoma City, OK United States; 2 Division of Hematology Department of Medicine Duke University Durham, NC United States; 3 Division of Blood Disorders National Center on Birth Defects and Developmental Disabilities Centers for Disease Control and Prevention Atlanta, GA United States

**Keywords:** venous thromboembolism, public health surveillance, machine learning, natural language processing, medical imaging review, public health

## Abstract

**Background:**

Venous thromboembolism (VTE) is a preventable, common vascular disease that has been estimated to affect up to 900,000 people per year. It has been associated with risk factors such as recent surgery, cancer, and hospitalization. VTE surveillance for patient management and safety can be improved via natural language processing (NLP). NLP tools have the ability to access electronic medical records, identify patients that meet the VTE case definition, and subsequently enter the relevant information into a database for hospital review.

**Objective:**

We aimed to evaluate the performance of a VTE identification model of IDEAL-X (Information and Data Extraction Using Adaptive Learning; Emory University)—an NLP tool—in automatically classifying cases of VTE by “reading” unstructured text from diagnostic imaging records collected from 2012 to 2014.

**Methods:**

After accessing imaging records from pilot surveillance systems for VTE from Duke University and the University of Oklahoma Health Sciences Center (OUHSC), we used a VTE identification model of IDEAL-X to classify cases of VTE that had previously been manually classified. Experts reviewed the technicians’ comments in each record to determine if a VTE event occurred. The performance measures calculated (with 95% CIs) were accuracy, sensitivity, specificity, and positive and negative predictive values. Chi-square tests of homogeneity were conducted to evaluate differences in performance measures by site, using a significance level of .05.

**Results:**

The VTE model of IDEAL-X “read” 1591 records from Duke University and 1487 records from the OUHSC, for a total of 3078 records. The combined performance measures were 93.7% accuracy (95% CI 93.7%-93.8%), 96.3% sensitivity (95% CI 96.2%-96.4%), 92% specificity (95% CI 91.9%-92%), an 89.1% positive predictive value (95% CI 89%-89.2%), and a 97.3% negative predictive value (95% CI 97.3%-97.4%). The sensitivity was higher at Duke University (97.9%, 95% CI 97.8%-98%) than at the OUHSC (93.3%, 95% CI 93.1%-93.4%; *P*<.001), but the specificity was higher at the OUHSC (95.9%, 95% CI 95.8%-96%) than at Duke University (86.5%, 95% CI 86.4%-86.7%; *P*<.001).

**Conclusions:**

The VTE model of IDEAL-X accurately classified cases of VTE from the pilot surveillance systems of two separate health systems in Durham, North Carolina, and Oklahoma City, Oklahoma. NLP is a promising tool for the design and implementation of an automated, cost-effective national surveillance system for VTE. Conducting public health surveillance at a national scale is important for measuring disease burden and the impact of prevention measures. We recommend additional studies to identify how integrating IDEAL-X in a medical record system could further automate the surveillance process.

## Introduction

Venous thromboembolism (VTE), which includes both deep vein thrombosis (DVT) and pulmonary embolism, is a common yet preventable vascular disease. The disease burden of VTE could be decreased through a coordinated approach to risk assessment, prophylaxis, and treatment [1]. In the United States, 36% to >50% of VTEs are associated with recent hospitalization or surgery and are considered hospital-associated VTEs [2-5]; therefore, hospital systems have the potential to facilitate effective VTE surveillance.

Conducting traditional VTE surveillance by using either active or passive methods is challenging because International Classification of Diseases codes for identifying VTE have been shown to have moderate sensitivity and positive predictive value [6-8], the manual review of medical records is labor intensive, and data entry is subject to human error. In the United States, the majority of newly generated clinical data are stored and analyzed digitally, typically in the form of an electronic medical record (EMR). As of 2017, EMRs are being used by 96% of nonfederal acute care hospitals [9], and EMR use has more than doubled since 2008 [10].

Despite years of progress in developing new database and file formats for medical record keeping, the majority of medical data are stored as unstructured text [3]. Unstructured text is a rich source of data for clinical and translational research [4]. Natural language processing (NLP) tools can be used to overcome the challenges of traditional VTE surveillance, as they can access the critical unstructured text from diagnostic imaging reports (eg, ultrasound and computed tomography [CT] angiography reports) [11], identify patients who meet the VTE case definition, and enter the relevant information into a surveillance database in an efficient amount of time [11-14].

Some of the key features involved with the use of NLP include preprocessing [7], syntactic processing, and concept and named entity recognition [6]. Preprocessing allows an algorithm to remove formatting (including carriage returns and other white-space characters) and then output a single “clean” string of text (free of markup or control characters pertaining to its original source) for later steps. Syntactic processing refers to understanding word order (eg, the subject-verb-object relationship) and references to vague nouns and pronouns, such as *it*. As a result, the algorithm is able to connect elements of complex or coordinated phrases. For example, in the sentence *There is no evidence of a filling defect in the right pulmonary artery*, the keywords that the algorithm needs to detect are *no*, *filling defect*, and *pulmonary artery*. Finally, concept and named entity recognition refer to the ability to identify variations in spelling or wording that relate to a single concept, such as the different ways clinicians may refer to, spell, or misspell *venous thromboembolism*. Linking different textual surface realizations (eg, *thrombus*, *embolism*, and *pulmonary embolism*) to a single conceptual entity (*venous thromboembolism*) facilitates classification and decreases the total number of parameters that need to be estimated in the model training stage.

Although the field of NLP is immense, with an ever-growing range of features and capabilities, the application of NLP in VTE surveillance is narrow. A specific software—IDEAL-X (Information and Data Extraction using Adaptive Learning; Emory University)—was used in a previous study to identify VTE by using the unstructured text from imaging records [14]. IDEAL-X leverages machine learning–based approaches to customize fine-tuned NLP models for various use cases. It analyzes domain-specific terminology and related linguistic features to determine a medical event. The IDEAL-X NLP tool has been applied to different use cases, and its applicability to VTE event identification has been proven by an Emory University pilot study [14]. When the IDEAL-X VTE identification model’s performance in the prefiltering of VTE records was tested in its native clinical setting, it demonstrated a sensitivity of ≥97.2% and a specificity of ≥99.3% [14]. However, since the NLP model was trained based on the records from an individual site, the prefiltering (eg, the identification of cases based on the type and severity of patients) and certain external factors (eg, speech patterns and word choices that are common to a certain clinic or geographic region) may have affected the performance of the NLP tool. Therefore, independent validation is required.

In order to evaluate the robustness and adaptability of our VTE identification model, which we developed based on the machine learning–based NLP tool IDEAL-X, and to determine how the differences among clinical settings can affect its performance (as a proof of concept for applying NLP to national VTE surveillance), we evaluated the accuracy of the VTE model in two independent health care settings—one in Durham, North Carolina, and another in Oklahoma City, Oklahoma.

## Methods

### Study Design

**Figure 1:**
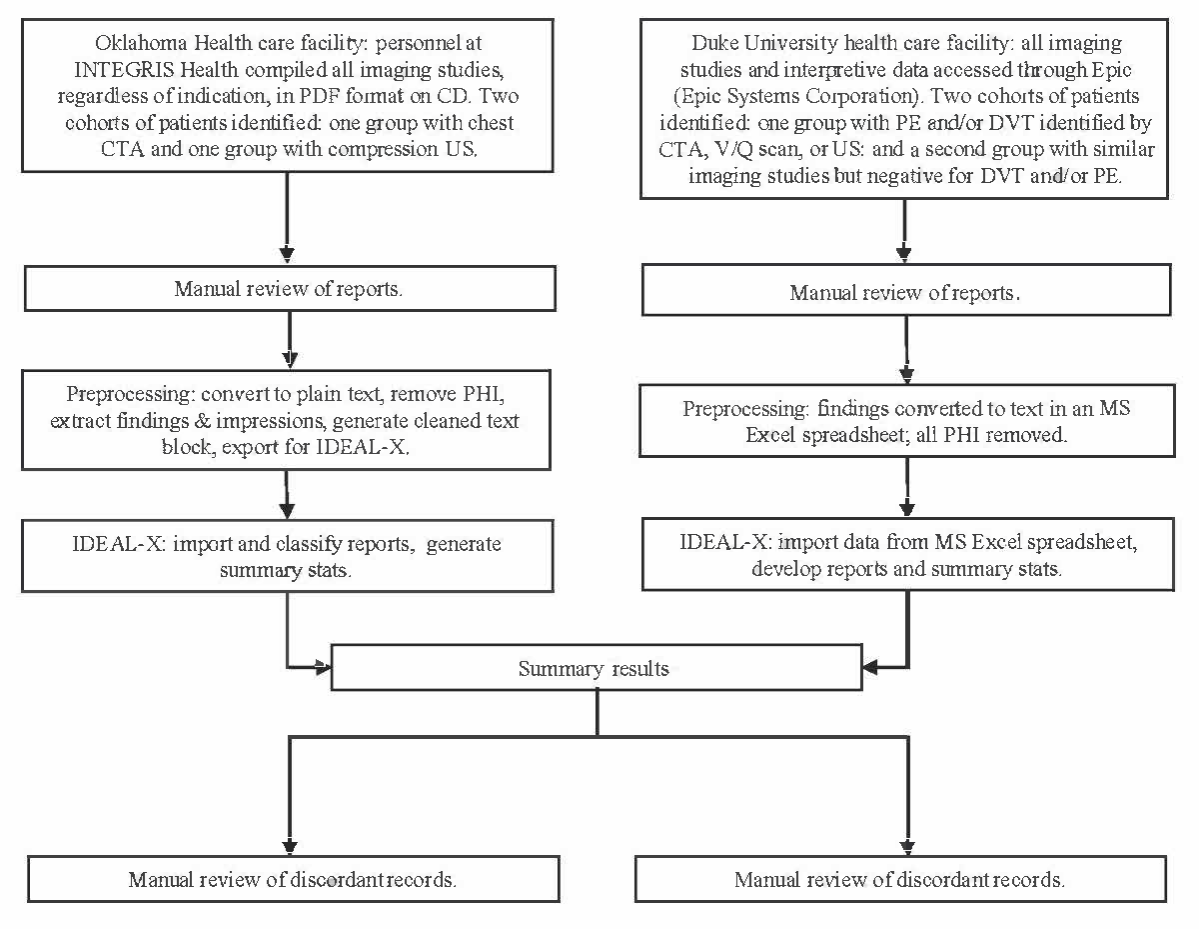
Flowchart of information collection and analysis at Duke University and the University of Oklahoma Health Sciences Center. CTA: computed tomography angiography; DVT: deep vein thrombosis; IDEAL-X: Information and Data Extraction Using Adaptive Learning; MS: Microsoft; PE: pulmonary embolism; PHI: personal health information; US: ultrasound; V/Q: ventilation/perfusion.

Duke University and the University of Oklahoma Health Sciences Center (OUHSC) collaborated with the Centers for Disease Control and Prevention to establish pilot surveillance systems for VTE [15,16]. The surveillance period (ie, for data collection) for both systems ranged from April 1, 2012, to March 31, 2014 (24 months). We used data from both surveillance systems for this study and evaluation. Members of each surveillance team served as the gold standard, manually reviewing imaging records and classifying them according to case status. Two investigators from the Duke University study team (IS and TO) and three investigators from the OUHSC study team (AW, NF, and GR) reviewed each record and classified them as positive or negative imaging reports of a DVT or pulmonary embolism. Subsequently, these records were “read” by IDEAL-X, which independently classified them according to case status. We evaluated the performance of the VTE model by comparing the case status results to the gold standard (manual review) findings. Site-specific details are described in the *Participants and Procedures* section, and the data collection and case classification methods are summarized in Figure 1.

### Ethical Considerations

This study was reviewed by the Duke University Institutional Review Board and the OUHSC Institutional Review Board. Both entities determined that this study did not include research on human subjects and was therefore exempt from institutional review board approval.

### Participants and Procedures

#### Duke University

The investigators at Duke University used the data set generated from the VTE surveillance program at three hospitals in Durham County, North Carolina (Duke University Hospital, Duke Regional Hospital, and the Durham Veterans Affairs Medical Center). The data set included all 818 unique records that were independently positive for the diagnosis of acute DVT, pulmonary embolism, or both (meeting the surveillance system’s case definition). To identify a total of 773 unique negative imaging records, the investigators reviewed (1) the negative imaging records from the same cohort of patients who also had a positive imaging study (eg, a negative lower extremity ultrasound from a patient with a positive CT angiogram) and (2) the negative imaging records from patients who were identified through the VTE surveillance program but were determined via the manual evaluation of the records to not have DVT or pulmonary embolism. The Duke University team manually extracted the findings and conclusions or the *Impression* sections from each imaging report to Microsoft Excel, regardless of the terminology or the contextual information. The team excluded additional text that described patient-specific information, the indication for the imaging study, and the type of imaging study used, as well as signature lines.

The radiographic imaging records included in the Duke University data set consisted of (1) ultrasound images of upper extremities, (2) ultrasound images of lower extremities, (3) CT angiography scans of the chest, and (4) ventilation-perfusion scans.

#### The OUHSC

The investigators at the OUHSC requested all of the imaging records from CT angiograms and compression ultrasounds, regardless of indication, from INTEGRIS Baptist Medical Center and INTEGRIS Southwest Medical Center. To our knowledge, the records were randomly selected and were representative of the patient population. This resulted in a data set with 1487 unique patients. The OUHSC team converted the PDF imaging records (ultrasound and CT records) to plain text format. We then used a search algorithm that was customized to the formatting conventions of the records to automatically locate and demarcate the *Impressions* and *Findings* sections. For each patient, these sections were extracted; cleaned of miscellaneous punctuation, white-space, and formatting characters; and converted into a text field for entry into the IDEAL-X package. Additional text processing was conducted to categorize records according to imaging type. All automated text processing at the OUHSC study site was performed by using Python v3.7.

#### The IDEAL-X Tool

The VTE identification model of IDEAL-X that we used in this analysis had been used in a previous study at Emory University [14]. In that study, IDEAL-X was used to analyze the radiology reports from the Emory University Orthopedic and Spine Hospital, which were dated from February 1, 2009, to December 9, 2014. The imaging reports included interpretations from ultrasound images of the lower and upper extremities, CT scans of the chest with contrast, and magnetic resonance images of the chest [14]. We applied the VTE identification model developed by the Emory project to our data sets as part of this study, without the further calibration or retraining of the model.

Both study sites (Duke University and the OUHSC) converted their data into the format required by IDEAL-X, which consisted of a Microsoft Excel spreadsheet containing the following four columns for data entry: the *ID*, *Text*, *Manual*, and *System* columns. The *ID* column contained a deidentified record ID that was computed from the PDF image file name by using a cryptographically secure hash function. The *Text* column contained the unstructured text that was extracted from the imaging reports after preprocessing. The *Manual* column contained the gold standard diagnosis for comparison with IDEAL-X results. The *System* column, per the IDEAL-X specification, was left blank and then populated with the automated classification after processing.

Additional aggregate outputs from IDEAL-X included the total number of records, the sensitivity, the specificity, the number of true and false positives, and the number of true and false negatives. Further, 95% CIs were calculated by using the Clopper-Pearson method for binomially distributed data [17]. Chi-square tests of homogeneity were conducted to evaluate differences in performance measures by site, using a significance level of .05. We conducted a post hoc analysis of the false-positive results, in which each coauthor reviewed the text of every false-positive and false-negative result and assigned it to one of the following categories: no evidence for thrombosis, superficial vein thrombosis, chronic or residual vein thrombosis, and indeterminate.

## Results

Duke University collected a total of 1591 imaging records (ultrasound images of upper extremities: n=223; ultrasound images of lower extremities: n=729; CT angiography scans of the chest: n=527; ventilation-perfusion scans: n=112). The OUHSC collected a total of 1487 imaging records (compression ultrasound images: n=1333; CT angiography scans of the chest: n=149; ventilation-perfusion scans: n=5). This provided our team with a combined total of 3078 records to be evaluated by IDEAL-X. The number of imaging records that IDEAL-X included or excluded (per the case definition for VTE) and the number of records that were manually reviewed are presented in Table 1 (the combined numbers and the numbers stratified by sites are shown). When both sites were aggregated, there were 1204 true-positive cases, 147 false-positive records, 1681 true-negative records, and 46 false-negative cases. The performance measures of the system are summarized in Table 2. Overall, the VTE model of IDEAL-X achieved over 90% accuracy (93.7%), sensitivity (96.3%), and specificity (92%).

When stratified by site, we found statistically significant differences in the performance measures between Duke University and the OUHSC. The sensitivity was significantly higher at Duke University (*P*<.001), while specificity was significantly higher at the OUHSC (*P*<.001). To further investigate differences in specificity, we identified the total number of false-positive results (147/1351, 10.9%). The reasons for the false-positive results are summarized in Table 3. The distribution varied between the two sites, and the categorical reason for false-positive results at Duke University was related to text indicating “there was no evidence for thrombosis” (104/104, 100%). Further, 38 of the 104 (36.5%) false-positive results at Duke University were from reports on ventilation-perfusion scans—an imaging modality that had not been included in the machine learning phase of the VTE identification model of IDEAL-X. The remaining errors occurred with the diagnostic imaging modalities that were previously used with the model (compression ultrasound and CT angiography), and many of the errors in the corresponding imaging reports were due to incorrect line breaks in the original text, which caused the algorithm to interpret the text incorrectly. In contrast, at the OUHSC, the most common reason for a false-positive result was text stating “a blood clot in a superficial vein” (25/43, 58.1%). The 38 false-positive results at Duke University from ventilation-perfusion scans represented 79.2% (38/48) of all ventilation-perfusion scans that were manually interpreted as *negative* at Duke University. In contrast, 20 of the 104 (19.2%) false-positive results at Duke University were from CT angiograms, but these represented only 8.1% (20/248) of all CT angiograms that were manually interpreted as *negative* at Duke University.

**Table 1:** The distribution of imaging records that the IDEAL-X (Information and Data Extraction Using Adaptive Learning) system identified as meeting the case definition for venous thromboembolism compared to the distribution of those identified via manual review (the gold standard). The combined distributions and the distributions stratified by surveillance site are shown.

Case classification		Classification via manual review								
		Combined			Duke University			The OUHSC^a^		
		Case, n	Noncase, n	Total classifications, N	Case, n	Noncase, n	Total classifications, N	Case, n	Noncase, n	Total classifications, N
**Overall classification^b^**										
	Case identified by IDEAL-X	1204	147	1351	801	104	905	403	43	446
	Noncase identified by IDEAL-X	46	1681	1727	17	669	686	29	1012	1041
	Total classifications by IDEAL-X	1250	1828	3078	818	773	1591	432	1055	1487
**Classification from compression ultrasound records**										
	Case identified by IDEAL-X	736	85	821	465	46	511	271	39	310
	Noncase identified by IDEAL-X	28	1436	1464	10	431	441	18	1005	1023
	Total classifications by IDEAL-X	764	1521	2285	475	477	952	289	1044	1333
**Classification from chest computed tomography angiogram records**										
	Case identified by IDEAL-X	403	24	427	274	20	294	129	4	133
	Noncase identified by IDEAL-X	15	234	249	5	228	233	10	6	16
	Total classifications by IDEAL-X	418	258	676	279	248	527	139	10	149
**Classification from ventilation-perfusion scan records**										
	Case identified by IDEAL-X	65	38	103	62	38	100	3	0	3
	Noncase identified by IDEAL-X	3	11	14	2	10	12	1	1	2
	Total classifications by IDEAL-X	68	49	117	64	48	112	4	1	5

^a^OUHSC: University of Oklahoma Health Sciences Center.

^b^Includes 112 ventilation-perfusion scans from Duke University and 5 ventilation-perfusion scans from the University of Oklahoma Health Sciences Center.

**Table 2:** The performance of the IDEAL-X (Information and Data Extraction Using Adaptive Learning) system by surveillance site.

Performance measure			Combined performance, % (95% CI)		Performance at Duke University, % (95% CI)		Performance at the OUHSC^a^, % (95% CI)
**Overall classification**							
	Accuracy	93.7 (93.7-93.8)		92.4 (92.3-92.5)		95.2 (95.1-95.2)	
	Sensitivity	96.3 (96.2-96.4)		97.9 (97.8-98)		93.3 (93.1-93.4)	
	Specificity	92 (91.9-92)		86.5 (86.4-86.7)		95.9 (95.8-96)	
	PPV^b^	89.1 (89-89.2)		88.5 (88.4-88.6)		90.4 (90.1-90.5)	
	NPV^c^	97.3 (97.3-97.4)		97.5 (97.4-97.6)		97.2 (97.1-97.3)	
**Classification from compression ultrasound records**							
	Accuracy	95.1 (95-95.1)		94.1 (94-94.2)		95.7 (95.6-95.8)	
	Sensitivity	96.3 (96.2-96.4)		97.9 (97.7-98)		93.8 (93.5-94)	
	Specificity	94.4 (94.3-94.5)		90.4 (90.1-90.5)		96.3 (96.2-96.3)	
	PPV	89.7 (89.5-89.8)		91 (90.8-91.1)		87.4 (87.1-87.7)	
	NPV	98.1 (98-98.1)		97.7 (97.5-97.9)		98.2 (98.1-98.3)	
**Classification from chest computed tomography angiogram records**							
	Accuracy	94.2 (94.1-94.3)		95.3 (95.1-95.4)		90.6 (90-91)	
	Sensitivity	96.4 (96.2-96.5)		98.2 (97.9-98.4)		92.8 (92.2-93.2)	
	Specificity	90.7 (90.3-91)		91.9 (91.6-92.2)		60 (53.9-65.4)	
	PPV	94.4 (94.2-94.5)		93.2 (92.9-93.4)		97 (96.4-97.3)	
	NPV	94 (93.6-94.2)		97.9 (97.5-98.1)		37.5 (34-41.6)	
**Classification from ventilation-perfusion scan records**							
	Accuracy	65 (64.2-65.6)		64.3 (63.5-65)		80 (67.4-87.9)	
	Sensitivity	95.6 (94.5-96.2)		96.9 (95.7-97.5)		75 (60-85.1)	
	Specificity	22.5 (21.3-24)		20.8 (19.7-22.4)		100 (47.5-100)	
	PPV	63.1 (62.3-63.8)		62 (61.2-62.8)		100 (78-100)	
	NPV	78.6 (73.7-82)		83.3 (77.6-87)		50 (27.5-72.5)	

^a^OUHSC: University of Oklahoma Health Sciences Center.

^b^PPV: positive predictive value.

^c^NPV: negative predictive value.

We also reviewed the false-negative results and summarized the findings in Table 3. Some of the potential reasons why IDEAL-X misclassified records could have been that (1) our manual reviewers had a lower threshold for investigating possible cases, such as classifying imaging records indicative of chronic VTE, a partially occluded blood vessel, or a diagnosis of thrombophlebitis as preliminary cases of VTE that would be further investigated and potentially ruled out upon further examination; (2) if the text indicated both evidence for a thrombus in one section and no evidence in another section, IDEAL-X deferred to the section indicating no evidence; and (3) IDEAL-X did not recognize certain misspellings or symbols. However, for 18 of the 46 (39.1%) false-negative cases, it is unclear why IDEAL-X misclassified the records. Of the 6 misclassified results at Duke University, 2 (33%) were from ventilation-perfusion scans.

**Table 3:** Reasons for discordant records.

Reasons in text		Duke University records, n (%)	OUHSC^a^ records, n (%)
**False-positive records**			
	No evidence for thrombosis	104 (100)	4 (9.3)
	Superficial vein thrombosis	0 (0)	25 (58.1)
	Chronic or residual deep vein thrombosis	0 (0)	13 (30.2)
	Indeterminate	0 (0)	1 (2.3)
	Subtotal	104 (100)	43 (100)
**False-negative records**			
	Inclusion of questionable cases as “positive”	2 (11.8)	9 (31)
	Positive and negative results in same report	2 (11.8)	6 (20.7)
	Unrecognized text or symbols, misspellings	7 (41.2)	2 (6.9)
	Positive report misclassified	6 (35.3)	12 (41.4)
	Subtotal	17 (100)	29 (100)

^a^OUHSC: University of Oklahoma Health Sciences Center.

## Discussion

### Principal Findings

This study suggests that IDEAL-X is an accurate NLP tool that can be used to identify cases of VTE. This system will likely improve the efficiency of VTE surveillance by automating the identification of VTE cases via accessing information from imaging records—the most reliable data source for VTE diagnosis. Our study results contribute to those published by Dantes et al [14] by broadening the scope of use from a specialty orthopedic hospital and demonstrating IDEAL-X’s utility and accuracy in general hospital settings within two different states with radiologists who used somewhat different language, word, and phrase patterns when interpreting imaging studies. In order to examine the robustness of the IDEAL-X VTE model, no additional training was applied subsequent to its configuration by researchers at Emory University [14]. Therefore, this study more fully explores the effect of how differences in hospital systems impact the VTE model’s performance.

The performance of such an NLP model was impacted by the imaging modality used. The specificity and positive predictive value for ventilation-perfusion scans, of which 95.7% (112/117) were collected from the Duke University system, were low. The specificity and negative predictive value of chest CT angiograms from the OUHSC were low. These values were likely impacted because we did not receive the requested sample (as demonstrated by only having 10 records from noncases). This resulted in a case prevalence of 93.2% (139/149), which is not representative of the prevalence of pulmonary embolism in the participating health system.

A particular advantage of using NLP to classify cases is the time required for IDEAL-X to classify the records according to case status. The preprocessing time for the OUHSC records (N=1487) was approximately 5 minutes, and the postprocessing time was <1 minute. In contrast, it takes approximately 1 minute per imaging study for a surveillance officer to read the text and classify it according to case status, which translates into potentially 52.5 person-hours for classifying the records used in this study. The time savings become increasingly meaningful when considering implementing surveillance across many facilities for a continuous time frame.

### Comparison With Prior Work

IDEAL-X is relatively simple compared to other common NLP tools, including cTAKES (Clinical Text Analysis Knowledge Extraction System), MetaMap, MedLEE (Medical Language Extraction and Encoding System), GATE (General Architecture for Text Engineering), NLTK (Natural Language Toolkit), and OpenNLP. Given that surveillance systems for VTE that use NLP are in a nascent stage of design and implementation, we have not yet included advanced features, such as coreference resolution, relation extraction, and semantic processing. However, these features may be warranted if additional detail is needed to identify physicians’ affiliations and organizations’ locations or to understand text that is as long as a paragraph (as opposed to 1-2 sentences).

In addition to being used in VTE case identification, IDEAL-X has also been used to extract treatment and prognosis information for patients with non–small cell cancer who are undergoing radiotherapy [18]; cardiac catheterization procedure reports; coronary angiography reports; and reports that contain unstructured text from medical histories, physicals, and hospital discharge summaries [19]. These studies report promising preliminary findings, showing precision values, sensitivity values, and *F* scores of 83% or greater.

Other NLP algorithms have been developed and used to identify cases of VTE. Hinz et al [20] developed an algorithm that reported a positive predictive value of 84.7%, a sensitivity of 95.3%, and an *F* score of 0.897. Gálvez et al [21] developed an NLP tool—Reveal NLP—that identified VTE cases in a pediatric population. The reported sensitivity was 97.2%, and the specificity was 92.5%. Although these previous studies used tools that they had developed, our study implemented IDEAL-X in institutions with no connection to the software’s development, providing additional insight into the usefulness and accuracy of the NLP tool.

### Limitations

A primary limitation of IDEAL-X is the lack of integration into an EMR system; IDEAL-X requires personnel to manually pull imaging records—a rate-limiting step. Another limitation is the forced binary options of *case* and *not a case*, such that *indeterminate* was not an option. The observed different distributions of categories of false-positive results by site were attributed to differences in the way records were requested or pulled at each site. Imaging studies from patients with superficial vein thrombosis and chronic or residual DVT were not included in the data set at Duke University. Enabling fast and convenient customization to support various event determination criteria would be a prerequisite for the NLP tool if nationwide deployment is required. In addition, further training is needed, so that IDEAL-X accurately classifies records in a manner that accounts for the patterns detected in false-positive and false-negative records. On the other hand, for surveillance purposes, the VTE case identification criteria also need to be standardized to ensure the consistency of case reporting among different facilities.

Future efforts will be directed at fully automating VTE surveillance. One example of how to better integrate an NLP program, such as IDEAL-X, is to include it in a facility’s clinical data process, so that after an imaging report is finalized and sent for billing, it is also run through IDEAL-X (and the associated preprocessing routines). In addition to classifying VTE cases in real time, the next step toward fully automating the process entails collecting demographic, clinical, and risk factor data to facilitate the interpretation of data regarding disease incidence. Other future efforts include implementing machine learning to fine-tune the IDEAL-X algorithm, so that it can “learn” how to more accurately differentiate between cases and noncases. Example text from records that generate false-positive results can be added to further train IDEAL-X and improve its accuracy. Despite the anticipated benefits of using these information extraction software tools, there are certain barriers to implementation. These barriers include the costs of customized deployment and localization and the proprietary nature of the software, as well as having personnel who are responsible for operating and maintaining the system, ensuring health care administrators buy into the benefits, and maintaining compliance with the Health Insurance Portability and Accountability Act and other regulations.

### Conclusions and Public Health Impact

The use of machine learning and NLP in disease surveillance is improving the ability to access and analyze unstructured text from EMRs. Their further and extensive use are expected to reduce resource requirements (ie, time and money), while increasing the ability to standardize data collection across sites. By conducting surveillance for VTE, we would have better data for knowing if changes in clinical practice (eg, an increase in the use of direct oral anticoagulants) are reducing the burden of VTE. Enhanced VTE surveillance can improve patient management, care, and safety. Similarly, with the advent of the COVID-19 pandemic, a robust national surveillance system would be instrumental in quickly understanding the association between COVID-19 and VTE [22]. The lessons learned from using NLP in VTE disease surveillance can be extended to improve the surveillance of other hospital-related conditions for which unstructured text from medical records plays a key role in detection and classification.

## Abbreviations

CT: computed tomography
cTAKES: Clinical Text Analysis Knowledge Extraction System
DVT: deep vein thrombosis
EMR: electronic medical record
GATE: General Architecture for Text Engineering
IDEAL-X: Information and Data Extraction Using Adaptive Learning
MedLEE: Medical Language Extraction and Encoding System
NLP: natural language processing
NLTK: Natural Language Toolkit
OUHSC: University of Oklahoma Health Sciences Center
VTE: venous thromboembolism
